# Molecular Mechanisms Underlying Differences in Athletic Ability in Racehorses Based on Whole Transcriptome Sequencing

**DOI:** 10.3390/biology14101364

**Published:** 2025-10-05

**Authors:** Qiuping Huang, Wanlu Ren, Dehaxi Shan, Yi Su, Zexu Li, Luling Li, Ran Wang, Shikun Ma, Jianwen Wang

**Affiliations:** 1 College of Animal Science, Xinjiang Agricultural University, Urumqi 830052, China; 18582253807@163.com (Q.H.); 13201295117@163.com (W.R.); 18093792263@163.com (D.S.); 13335339131@163.com (Y.S.); 13593312012@163.com (Z.L.); 18996888638@163.com (L.L.); 17590811761@163.com (R.W.); 18299152719@163.com (S.M.); 2 Xinjiang Key Laboratory of Equine Breeding and Exercise Physiology, Urumqi 830052, China

**Keywords:** horse racing, whole transcriptome, Yili horse, locomotor ability, gene expression

## Abstract

**Simple Summary:**

This study conducted whole-transcriptome sequencing on Yili horses with outstanding and average performance in the 5000 m race, identifying multiple exercise-related differentially expressed genes, including EGR1, FOSB, MRPL1, LOC100049811, SIRPB2, and CYTB. These genes, along with their associated mRNAs, lncRNAs, and circRNAs, synergistically participate in energy metabolism, protein homeostasis, and muscle remodeling processes, collectively enhancing the endurance and recovery capacity of Yili horses in long-distance races. This study provides important references for the identification of candidate genes related to equine athletic performance.

**Abstract:**

This study aimed to compare blood samples from Yili horses with outstanding and average performance in 5000 m races through transcriptome sequencing, identify key differentially expressed genes, lncRNAs, and circRNAs, as well as related enriched pathways, and elucidate their regulatory networks. This study used six healthy four-year-old Yili stallions as subjects, divided into an excellent group (E group, *n* = 3) and an ordinary group (O group, *n* = 3) based on their 5000-m race performance. Blood RNA-Seq technology was used to analyze differentially expressed mRNAs, lncRNAs, and circRNAs. A total of 2298 mRNAs, 264 lncRNAs, and 215 circRNAs were identified as differentially expressed. Key genes such as EGR1, FOSB, MRPL1, LOC100049811, SIRPB2, and CYTB regulate athletic performance. These genes and their associated RNAs synergistically participate in energy metabolism, protein homeostasis, and muscle remodeling processes, revealing the molecular mechanisms influencing athletic performance and providing important references for identifying candidate genes associated with equine athletic performance.

## 1. Introduction

Currently, studies on equine athletic ability mainly focus on physiological indicators (e.g., heart rate, blood lactate concentration) [1,2], muscle structure, blood biochemical characteristics [3], and detecting a few known genes (e.g., MSTN, PPARGC1A, PDK4) [4,5,6]. However, the formation of equine athletic performance is a complex physiological process [7], in which genes and regulatory factors play an important role [5]. The frequency of the AA and AG genotypes at the G38973231A locus of the PDK4 gene was significantly higher than that of the GG genotype in both speed groups (*p* < 0.05) [6], but the regulatory mechanism has not yet been clarified.

Exercise can directly influence the transcription of key regulatory genes [8], metabolic genes, and myogenic genes. Current research focusing on mRNA has identified multiple key pathways associated with muscle development [9] and metabolic regulation [10]. Long non-coding RNAs (lncRNAs) and circular RNAs (circRNAs) are non-coding RNAs that influence diverse biological processes by regulating gene expression [11]. Studies indicate that lncRNAs play crucial roles in mammalian muscle development [12], energy metabolism [13], and stress responses [14]. Exercise-induced gene expression changes were identified in the blood and muscles of Arabian racehorses, providing transcriptomic insights into the molecular mechanisms of exercise adaptation [15]. Furthermore, analyses of ancient and modern horse genomes indicate that lncRNA-mediated epigenetic regulation significantly influenced horse domestication [16]. lncRNAs and circRNAs perform complex roles in equine exercise physiology, influencing gene expression and muscle development through diverse mechanisms such as regulating muscle tissue expression and participating in regulatory networks [17]. Further exploration of interactions between mRNAs and non-coding RNAs may yield novel strategies for enhancing equine athletic performance and health.

We hypothesize that there may be a potential association between racehorse performance and mRNA, long non-coding RNA (lncRNA), and circular RNA (circRNA). This study employed RNA-Seq on blood samples from high-performing and average-performing Yili horses to identify differentially expressed mRNAs, lncRNAs, and circRNAs, aiming to uncover their molecular mechanisms in athletic ability development. This study will not only establish a theoretical basis for improving the athletic ability and training of Yili horses but also offer a new research perspective for studying equine sports genomes.

## 2. Materials and Methods

This study was approved by the Animal Welfare and Ethics Committee of Xinjiang Agricultural University (approval number: 2024003).

### 2.1. Experimental Animals and Sample Collection

In this study, blood samples were collected from 24 Yili horses participating in the 5000 m race before the race. Blood samples (5 mL) were collected from the jugular vein of all horses using EDTA-anticoagulated vacuum tubes. The collected blood samples were stored in liquid nitrogen. Following the competition, blood samples from the top three performers (Excellent Group, E Group) and the bottom three performers (Ordinary Group, O Group) were selected based on competition results for transcriptomic analysis. All horses were professionally trained by their owners and underwent a pedigree check and a health check for lameness by a veterinarian before the race. Blood samples were collected 1 day before the race at 20:00 h. The selected Yili horses for testing were all stallions. Prior to sampling, the horses were kept under identical husbandry conditions and were all four years old. The race results were 5′23″704–5′41″339 for the excellent group and 7′43″247–8′36″489 for the ordinary group.

### 2.2. RNA Extraction and Quality Control, Library Construction, and High-Throughput Sequencing

Total RNA was extracted from horse blood samples in the E and O groups using TRNzol Universal Reagent (Tiangen, Beijing, China, Catalog No.: DP424) according to the manufacturer’s instructions. RNA purity and concentration were determined using a NanoDrop 2000 spectrophotometer (OD260/280 ratio between 1.8–2.1, Thermo Fisher Scientific, Carlsbad, CA, USA), and integrity was assessed by measuring the RNA Integrity Number (RIN) using an Agilent 2100 Bioanalyzer. Samples meeting RIN ≥ 7.0 and 28S/18S ≥ 1.8 criteria were used for subsequent experiments. Total RNA was extracted from the blood of horses in groups E and O using the Fast RNA-seq Lib Prep Kit V2 (Cat. No. RK20306, Vazyme Biotech, Thousand Oaks, CA, USA). rRNA was removed from the total RNA samples (some lncRNAs have the same poly A tail structure as mRNAs, and the method of removing rRNAs can maximize the retention of poly A tails in lncRNAs), and then a fragmentation buffer was added to the enriched RNAs to break the RNA into small fragments. Using the fragmented RNA as a template, the first strand of cDNA was synthesized using random hexamer primers and reverse transcriptase, and then the second strand of cDNA was synthesized by adding buffer and dNTPs (dTTP in dNTP was replaced by dUTP). The synthesized double-stranded cDNA was processed through end repair, A addition, and ligation of sequencing junctions, and the fragments were screened for PCR enrichment. To screen the cDNA fragments with a length of 370~420 bp, the PCR products were purified using AMPure XP beads, and the strand-specific libraries were finally obtained. AATI was used to detect the integrity of the library fragments and the size of the inserted fragments, and QPCR was used to detect the effective concentration of the libraries. After passing the library test, the different libraries were pooled according to the effective concentration and the target downstream data volume and then sequenced at both ends.

### 2.3. Differential Expression Gene Analysis

Sequencing-derived clean reads were first aligned to the reference genome (https://ftp.ncbi.nlm.nih.gov/genomes/all/GCF/002/863/925/GCF_002863925.1_EquCab3.0/, accessed on 24 May 2025) to obtain gene localization information. Mapping was performed using HISAT2 (v2.0.5). Subsequently, featureCounts was employed to count the mapped results, yielding a raw read count matrix at the gene or transcript level. Annotation information was sourced from the official genome annotation file (GFF3), encompassing mRNA, lncRNA, and circRNA.

Significant expression differences were analyzed using edgeR [18], with *p*-values or corrected *p*-values determining significance thresholds. Raw read counts were normalized primarily to correct for sequencing depth; negative binomial generalized linear model hypothesis testing probabilities (*p*-values) were then calculated; finally, multiple hypothesis testing correction yielded FDR values. Genes meeting the criteria of |log2(FC)| ≥ 1 and *p*-value < 0.05 were considered differentially expressed. circRNA detection and identification were performed using find_circ [19] and CIRI2 [20] to enhance accuracy.

Based on alignment results, Stringtie (v1.3.3) was used to assemble reads into transcripts and perform quantification [21]. The merge parameter in Stringtie was applied to combine transcripts assembled across samples, removing those with uncertain strand orientation and transcripts shorter than 200 nt. Transcripts were compared against known databases using gffcompare (v0.10.6), filtered for known transcripts, and subjected to coding potential prediction for newly identified transcripts. Expression levels were quantified for both aligned/assembled/filtered transcripts and predicted transcripts. lncRNA seq data were expressed using FPKM. Expression levels of known and novel circRNAs in each sample were statistically analyzed. Expression normalization was performed using TPM [22], calculated as normalized expression = (read count × 1,000,000)/libsize (where libsize is the total sum of circRNA read counts).

### 2.4. Functional Enrichment Analysis

Differentially expressed genes were analyzed for GO enrichment analysis with clusterProfiler. Significantly enriched GO terms were identified based on padj < 0.05 as the threshold for statistical significance. KEGG (Kyoto Encyclopedia of Genes and Genomes) is the primary public database for pathways (https://www.kegg.jp/kegg-bin/show_organism?org=ecb, accessed on 3 June 2025). Pathway significance enrichment analysis was conducted using KEGG pathways as units, applying the hypergeometric test to identify significantly enriched pathways among candidate target genes, with a significance threshold of padj < 0.05 (lncRNA significantly co-expressed (Pearson correlation coefficient > 0.9, padj < 0.05) and co-expressed within a 100 kb range upstream or downstream of the target gene set), and the enrichment of differentially expressed genes in KEGG pathways was analyzed using the clusterProfiler software. A *p*-value < 0.05 was used as the criterion for screening significantly enriched GO terms or KEGG pathways.

### 2.5. Protein Interaction Network Analysis

Combining differential expression analysis results with interaction pairs from databases, protein–protein interactions (PPIs) were constructed using the STRING database. Subsequently, an interaction network was built based on homologous protein interactions, visualized using Cytoscape software (v3.8.0), and the top 10 core genes of the protein interaction network were identified via the cytoHubba plugin.

## 3. Results

### 3.1. Transcriptome Quality Control Analysis

As shown in Table 1, the raw data from the blood transcriptomes of groups E and O comprised 290,350,788 and 243,884,046 reads, respectively. After quality control and filtering, 281,014,054 and 237,778,098 high-quality reads were obtained. The percentage of Q20 bases was higher than 98.1%, the percentage of Q30 bases was higher than 94.88%, and the GC content was between 46.09% and 49.91%. 46.09% to 49.91%. When comparing the clean reads to the horse reference genome, the average comparison efficiency of the six samples was 93.87%. This shows that the sequencing data are of excellent quality and meet the requirements for subsequent analysis.

### 3.2. Gene Expression Level Analysis

**Figure 1:**
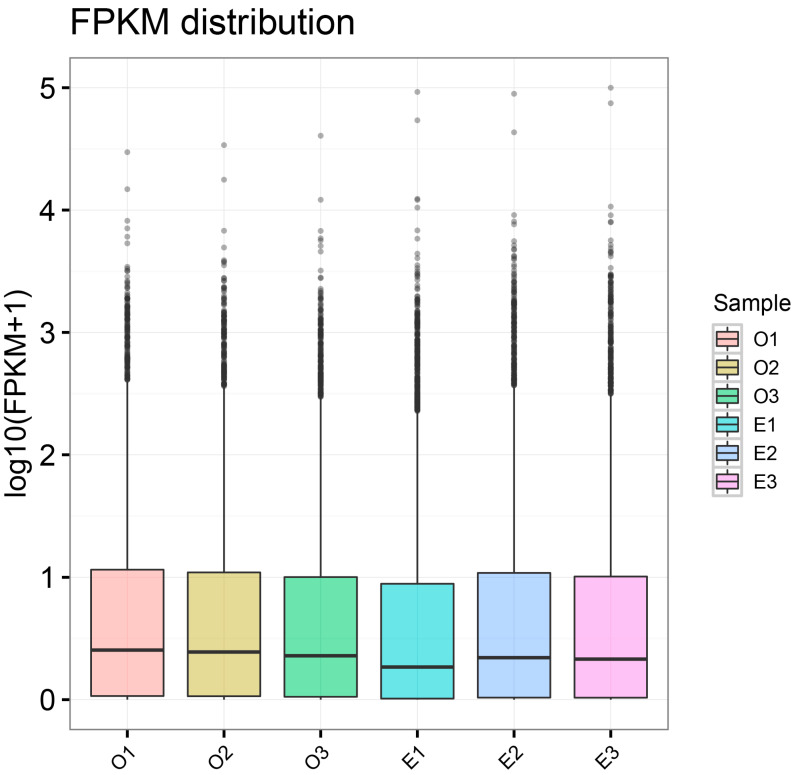
Distribution of Expression Levels Across Samples. Box plots of expression levels for different samples, where the *x*-axis represents sample names and the *y*-axis represents log10(FPKM + 1), the box indicates the interquartile range (IQR), and gray dots denote outliers exceeding 1.5 times the IQR. Each region’s box plot corresponds to five statistical measures (from top to bottom: maximum value, upper quartile, median, lower quartile, and minimum value).

After log10 transformation, the FPKM distributions of horse blood samples in Groups E and O exhibited highly consistent boxplot distributions. No significant differences were observed in overall gene expression levels between the ordinary group (O1–O3) and the excellent group (E1–E3). The medians of the six samples were close, the box ranges were similar, and the distribution of outliers in highly expressed genes was also more consistent (Figure 1). This indicates balanced sequencing quality among samples, good data standardization, and comparability for subsequent differential expression analysis.

### 3.3. Identification of Differentially Protein-Coding Genes and Statistical Identification of Differentially Non-Coding Genes

**Figure 2:**
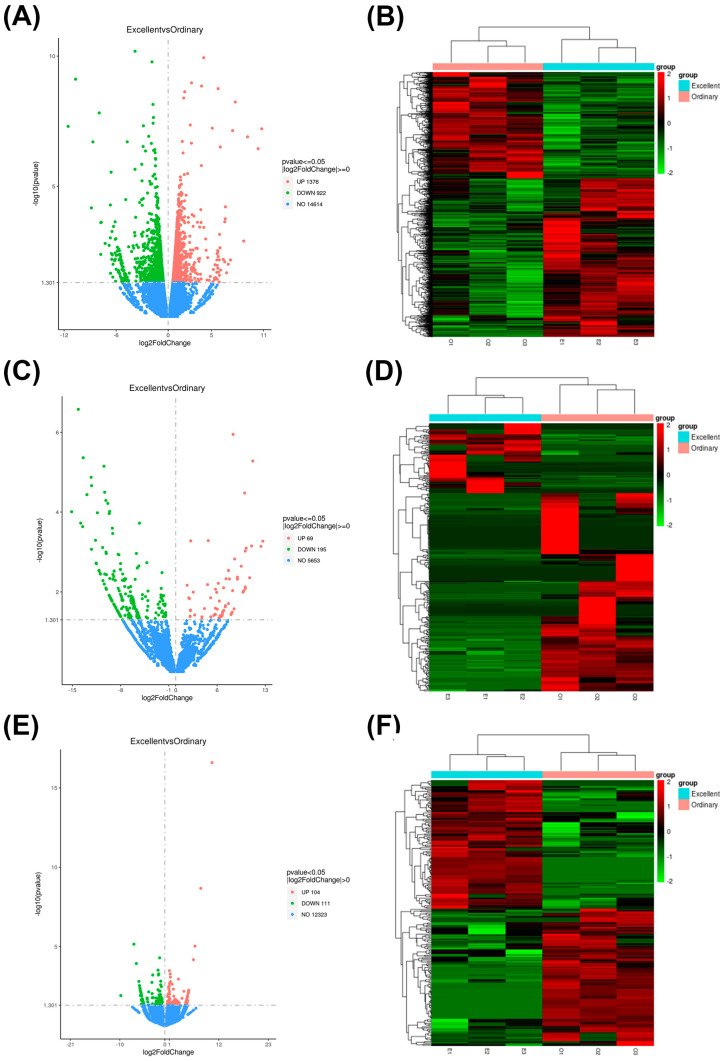
mRNA differential gene volcano plot (**A**), mRNA differential gene hierarchical clustering heat map (**B**), lncRNA differential gene volcano plot (**C**), lncRNA differential gene hierarchical clustering heat map (**D**), circRNA differential gene volcano plot (**E**), circRNA differential gene hierarchical clustering heat map (**F**). In the volcano plot, the *x*-axis represents the fold change (log2FoldChange) in mRNA/lncRNA/circRNA expression between high-performing and average-performing horses in the Yili horse race. A larger absolute value on the *x*-axis indicates a greater fold change in expression between the two comparison groups; the *y*-axis indicates the significance level of expression differences. The orange-yellow dashed line near *y*-axis 1.30 corresponds to qvalue (default) or pvalue = 0.05. Upregulated genes are represented by red dots, downregulated genes by green dots, and genes with no significant changes by blue dots. In the cluster analysis heatmap, the *x*-axis represents samples, and the *y*-axis represents differentially expressed genes. The left side clusters genes based on expression similarity, and the top clusters each sample based on expression profile similarity. Expression levels increase gradually from green to red, with numbers indicating the normalized relative expression levels.

In order to understand the effect of differential gene expression on Yili horse sports, we compared and analyzed the differentially expressed genes in two different groups with excellent performance in the 5000 m race E and average performance in race O. In Figure 1, the genes are identified as differentially protein-coding genes and differentially non-coding genes. By calculating and screening the significantly differentially expressed genes related to Yili horse sports, 2298 differentially expressed genes were identified in the mRNA of Yili horses with different race performance, of which 1376 were up-regulated and 922 were down-regulated (Figure 2A,B). The significantly up-regulated genes included EGR1, LOC111767503, FOSB, MRPL1, LOC100049811, SIRPB2, EQMHCC1, and the significantly down-regulated genes included CFAP43, ZCCHC7, LOC111771913. The analysis of differential expression of lncRNA genes with different races results in Yili horses showed that 264 of them were differentially expressed lncRNA genes, of which 69 were up-regulated and 195 were down-regulated(Figure 2C,D). A total of 215 differentially expressed circRNA genes were detected in horses with different race results, of which 104 genes were up-regulated and 111 genes were down-regulated (Figure 2E,F).

### 3.4. GO and KEGG Enrichment Analysis of Differentially Expressed mRNA Genes

**Figure 3:**
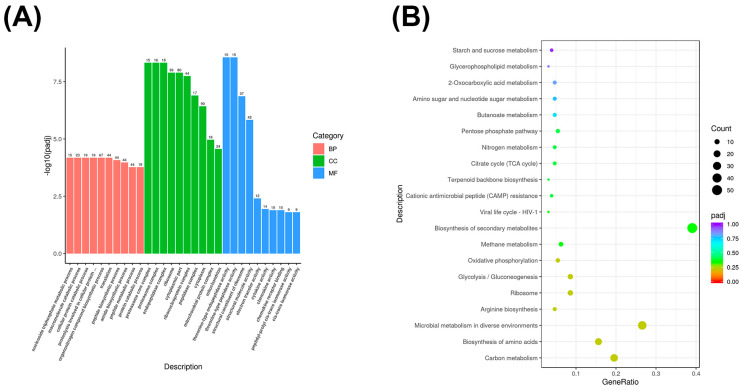
(**A**) mRNA GO enrichment bar chart. The *x*-axis represents GO terms, and the *y*-axis represents the significance level of GO term enrichment, expressed as −log10(padj) values. Different colors represent the three GO subcategories: biological process (BP), cellular component (CC), and molecular function (MF). (**B**) mRNA KEGG enrichment scatterplot. The *y*-axis represents different pathways, and the *x*-axis represents the proportion of significantly differentially expressed genes in a given pathway relative to all genes in that pathway. The size of the circle represents the number of genes enriched in a given pathway; the larger the circle, the more genes enriched in that pathway. Colors represent enrichment significance; the closer the color is to red, the higher the significance.

The functions and metabolic pathways of mRNA-differentially expressed genes were described by GO enrichment and KEGG enrichment analyses of significantly differentially expressed genes with different scores in 5000 m races of Yili horses. The differentially expressed genes in the excellent group (Group E) and the average group (Group O) were mainly enriched in the biological processes of nucleoside triphosphate metabolism (GO: 0009141), macromolecule catabolism (GO: 0009057), cellular proteolysis metabolism (GO: 0044257), and protein hydrolysis involved in cellular proteolysis metabolism (GO: 0051603). In cellular composition, they were mainly enriched in components such as proteasome core complex (GO: 0005839), proteasome complex (GO: 0000502), and endopeptidase complex (GO: 1905369) molecular functions, they were mainly enriched in functions such as threonine-type endopeptidase activity (GO: 0004298) and threonine-type peptidase activity (GO: 0070003) (Figure 3A).

KEGG enrichment analysis revealed that the differentially expressed mRNAs in groups E biosynthesis, microbial metabolism in different environments, arginine biosynthesis, ribosomes, and O were mainly enriched in the interactions between carbon metabolism, amino acid/glycolysis/glycolysis, and oxidative phosphorylation pathways (Figure 3B).

### 3.5. Co-Expressed Long Non-Coding RNA (lncRNA) Target Gene GO and KEGG Enrichment Analysis

**Figure 4:**
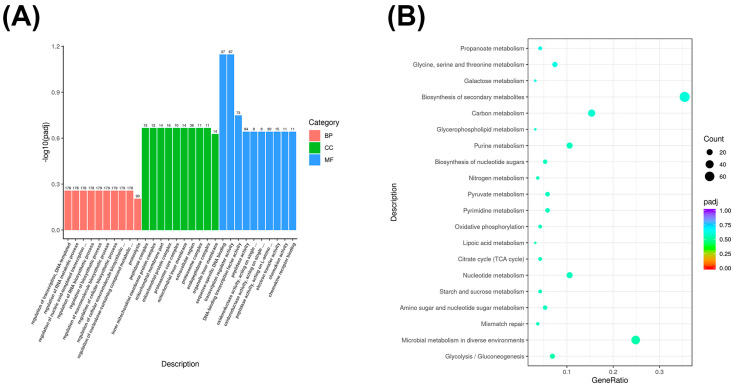
(**A**) GO enrichment bar chart of co-expressed long non-coding RNAs (lncRNAs). (**B**) KEGG enrichment scatter plot of co-expressed long non-coding RNAs (lncRNAs).

We analyzed the common long non-coding RNA (lncRNA) target genes in the different performance groups of the Yili horse in the GO and KEGG enrichment analyses. We described the functions of the common lncRNA target genes. The GO enrichment showed that lncRNA target genes in Groups E and O were enriched in the transcription regulation process with DNA as a template (GO: 0006355), RNA metabolism (GO:0051252), nucleic acid template transcription (GO:1903506), RNA biosynthetic process (GO:2001141), biosynthetic process (GO:0009889), macromolecular biosynthetic process (GO:0010556), cellular biosynthetic process (GO:0031326), and cellular macromolecular biosynthetic process (GO: 2000112), and the regulation of nucleoside compound metabolic process (GO:0019219). In cellular composition, they were primarily enriched in peptidase complex (GO:1905368) and mitochondrial inner membrane protein complex (GO:00988002), the mitochondrial membrane (GO:0044455), mitochondrial protein complexes (GO:0098798), the proteasome core complex (GO:0005839), the mitochondrial inner membrane (GO:0005743), the extracellular region (GO:0005576), the proteasome complex (GO:0000502), and the endopeptidase complex (GO:1905369). In terms of molecular function, they are primarily enriched in sequence-specific DNA binding (GO:0043565) and transcription regulatory activity (GO:0140110) (Figure 4A).

KEGG enrichment analysis revealed that co-expressed long non-coding RNA (lncRNA) target genes are primarily enriched in glycolysis/gluconeogenesis and microbial metabolism in different environments (Figure 4B).

### 3.6. Co-Localized Long Non-Coding RNA (lncRNA) Target Gene GO and KEGG Enrichment Analysis

**Figure 5:**
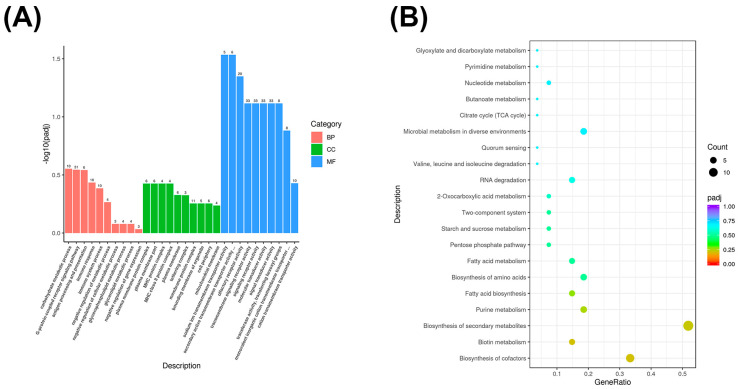
(**A**) GO enrichment bar chart of co-localized long non-coding RNAs (lncRNAs). (**B**) KEGG enrichment scatter plot of co-localized long non-coding RNAs (lncRNAs).

We analyzed the co-localized lncRNA target genes of Yili horses with different performance levels in the competition using GO enrichment and KEGG enrichment analysis and described the functions of the co-localized lncRNA target genes. GO enrichment results showed that the E and O groups of Yili horses with excellent and average performance in the competition were primarily enriched in carbohydrate metabolism processes (GO:0005975), G protein-coupled receptor signaling pathways (GO:0007186), and antigen processing and presentation (GO:0019882) in biological processes. In terms of cellular composition, they were primarily enriched in plasma membrane protein complexes (GO:0098797), plasma membrane components (GO:0044459), and major histocompatibility complex proteins (GO:0042611). In terms of molecular function, they are associated with plasma membrane protein complexes (GO:0098797), plasma membrane components (GO:0044459), major histocompatibility complex proteins (GO:0042611), sodium ion transmembrane transport activity (GO:0015081), secondary active transmembrane transport activity (GO:0015291), and olfactory receptor activity (GO:0004984) (Figure 5A).

KEGG enrichment analysis revealed that co-localized lncRNA genes are primarily enriched in cofactor biosynthesis and biotin metabolism (Figure 5B).

### 3.7. GO and KEGG Enrichment Analysis of Differentially Expressed circRNA Genes

**Figure 6:**
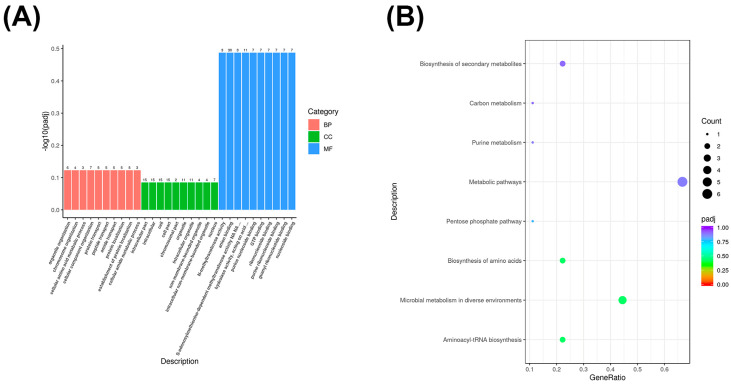
(**A**) GO enrichment bar chart of differentially expressed circRNA genes. (**B**) KEGG enrichment scatter plot of differentially expressed circRNA genes.

We performed GO enrichment and KEGG enrichment analysis on significantly differentially expressed genes in Yili horses with different performance levels in competitions to describe the functions and metabolic pathways of differentially expressed circRNA genes. Differentially expressed genes in Group E and Group O were primarily enriched in organelle organization within cellular composition (GO: 0006996), chromosome organization (GO: 0051276), cellular amino acid metabolism (GO: 0006520), cellular component organization (GO: 0016043), protein transport (GO: 0015031), peptide transport (GO: 0015833), amide transport (GO: 0042886), protein localization (GO: 0008104), establishment of protein localization (GO: 0045184), and cellular amide metabolism (GO: 0043603). In cellular components, they are primarily enriched in intracellular compartments (GO: 0044424), intracellular (GO: 0005622), cells (GO: 0005623), cellular components (GO: 0044464), chromosomal components (GO: 0044427), organelles (GO: 0043226), intracellular organelles (GO: 0043229), non-membrane-bound organelles (GO: 0043228), intracellular non-membrane-bound organelles (GO: 0043232), and the nucleus (GO: 0005634). Their primary molecular functions include N-methyltransferase activity (GO: 0008170), anion binding (GO: 0043168), S-adenosylmethionine-dependent methyltransferase activity (GO: 0008757), hydrolase activity acting on phosphoric acid esters (GO: 0016818), purine nucleoside binding (GO: 0001883), GTP binding (GO: 0005525), ribonucleotide binding (GO: 0032549), purine ribonucleotide binding (GO: 0032550), guanosine ribonucleotide binding (GO: 0032561), and nucleotide binding (GO: 0001882) (Figure 6A).

KEGG enrichment results showed that differentially expressed circRNAs were primarily enriched in metabolic pathways such as amino acid biosynthesis, microbial metabolism in diverse environments, and aminoacyl-tRNA biosynthesis (Figure 6B).

### 3.8. Protein Network Interactions Results Analysis

**Figure 7:**
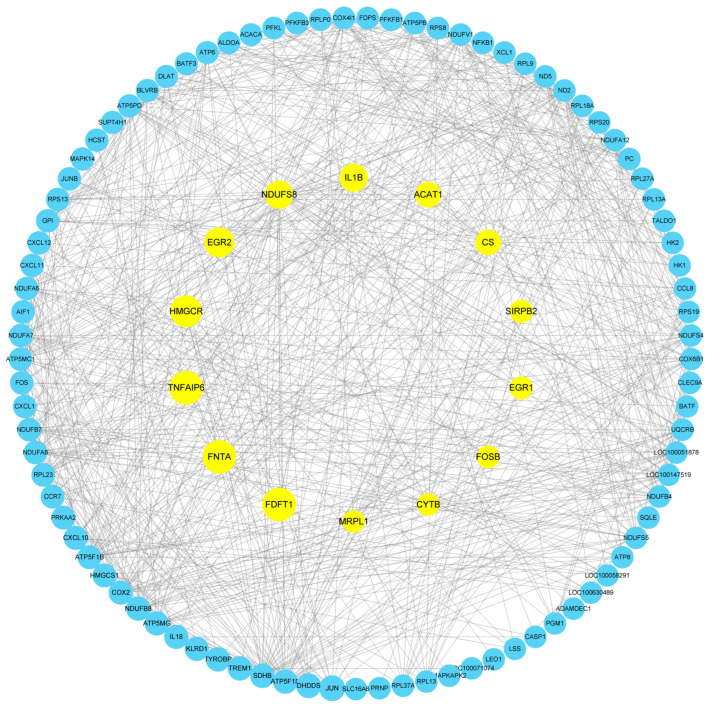
Protein Network Interaction Map of Differentially Expressed Genes: Interaction analysis of protein networks for Excellent and Ordinary differentially expressed genes. The outer blue nodes represent differentially expressed genes, while the inner yellow nodes denote core genes (*n* = 10) and key genes extracted in this study. The lines indicate significant co-expression relationships.

This study constructed a protein interaction network of differentially expressed genes in blood samples from Yili horses before and after competition, identifying core genes including FNTA, HMGCR, EGR2, IL1B, CS, SIRPB2, CYTB, and MRPL1 (Figure 7).

## 4. Discussion

The performance of racehorses—such as speed, endurance, and explosive power—is strongly influenced by genetic factors [23,24]. Whole-transcriptome analysis can help identify genes and metabolic pathways associated with racehorse athletic ability [7]. This study aimed to reveal the potential molecular mechanisms underlying the differences in athletic performance between the Yili horse and the ordinary horse during long-distance races through transcriptome-wide analysis. We identified differentially expressed mRNAs, lncRNAs, and circRNAs between the excellent group (E group) and ordinary group (O group). These differentially regulated genes and non-coding RNAs were predominantly enriched in key pathways including energy metabolism, protein processing, and immune response. Key genes significantly upregulated in the E group (e.g., EGR1, FOSB, MRPL, CYTB) are closely associated with skeletal muscle function and mitochondrial metabolism. These findings support the hypothesis that the mRNA–lncRNA–circRNA synergistic network may play a crucial role in maintaining energy supply, protein homeostasis, and immune adaptation.

Skeletal muscle function is crucial for exercise [25]. EGR1 promotes skeletal muscle satellite cell differentiation by regulating MyoG gene expression [26]. Exercise, as a physiological stimulus, can rapidly activate EGR1, thereby initiating a series of gene expression programs. These gene alterations ultimately influence adaptive responses in skeletal muscle [27], promoting muscle repair and regeneration [28]. They also regulate antioxidant gene expression to mitigate oxidative stress in muscle cells, protecting them from damage [29,30]. This aligns with the significant upregulation of EGR1 observed in the E group in this study.

FosB belongs to the AP-1 transcription factor family, which has been shown to regulate osteoblast differentiation and bone formation [31]. In mouse studies, FosB can be rapidly induced at the transcriptional level by mechanical stress [32]. Overexpression of the ΔFosB transcription factor can increase bone formation and inhibit fat production [33]. In this study, FOSB was significantly upregulated in the E group, indicating that exercise can influence FOSB expression and thereby affect osteoblast differentiation.

Mitochondria are the cell’s energy powerhouses, generating ATP through oxidative phosphorylation [34]. The MRPL gene encodes mitochondrial ribosomal proteins involved in mitochondrial protein synthesis [35]. Exercise requires substantial energy, making mitochondrial function critical for exercise performance. Mitochondrial dysfunction may lead to reduced exercise capacity and even related diseases [36]. Studies have shown that mutations in the MRPL gene are associated with various diseases, such as cardiomyopathy, psychomotor retardation, and respiratory chain defects [36]. Galmiche et al. found that different types of exercise (e.g., endurance and resistance training) and training states (e.g., untrained and trained) induce distinct gene expression changes, which may influence mitochondrial function and energy metabolism, thereby affecting athletic performance [37]. This is consistent with the upregulation of the MRPL gene observed in this study.

LOC100049811 primarily acts on PLAAT3 phospholipase A and acyltransferase 3. PLAAT3 primarily catalyzes the hydrolysis and acyltransferase reactions of phospholipids and may play a potentially important role in cell membrane dynamics, signal transduction, apoptosis, and metabolic regulation [38,39], and is involved in peroxisome-regulated plasmalogen metabolism and PUFA metabolism [40]. PUFAs (polyunsaturated fatty acids) are crucial for skeletal muscle function, influencing skeletal muscle health and function through multiple mechanisms, including regulating inflammatory responses, promoting muscle protein synthesis, and affecting the composition of muscle cell membranes [41,42]. PLAAT3 itself may influence muscle oxidative capacity by affecting the content of DHA (docosahexaenoic acid) in skeletal muscle cell membranes, modulating skeletal muscle adaptability through PPARδ activation, thereby impacting exercise endurance [43], and regulating inflammatory mediator production by modulating phospholipid metabolism, thereby participating in the regulation of exercise-induced inflammatory responses [44]. This study showed that in Group E, the LOC100049811 gene was upregulated, possibly acting on PLAAT3 to regulate skeletal muscle function, adaptability, and oxidative capacity and reduce exercise-induced inflammatory responses.

Signal-regulating protein β2 (SIRPB2) is a member of the signal-regulating protein (SIRP) family, expressed in immune cells, and participates in cell-to-cell interactions and signal transduction [45,46]. The SIRP family includes activating, inhibitory, and non-signaling variants, which share closely related extracellular regions but differ in their cytoplasmic tails. They are primarily expressed in myeloid cells and are therefore believed to play a role in immune regulation [47]. The interaction between SIRPB2 and CD47 can regulate inflammatory responses, cell migration, cell adhesion, and energy metabolism [48], thereby playing a role in tissue repair and reconstruction induced by exercise [49]. In this study, SIRPB2 was significantly upregulated, potentially affecting the migration and adhesion of immune cells, thereby regulating the recruitment of inflammatory cells to the injury site.

The cytochrome b (CYTB) gene plays a key role in the mitochondrial genome, with its encoded transmembrane protein participating in energy metabolism and individual exercise endurance. Mutations or dysfunction of the CYTB gene can impair the efficiency of the mitochondrial respiratory chain, thereby affecting cellular energy metabolism, leading to exercise intolerance and alterations in training adaptation. Exercise training can regulate CYTB expression by stimulating mitochondrial biogenesis and epigenetic modifications, thereby enhancing energy metabolism efficiency and exercise performance. The CYTB gene encodes a 513-amino-acid transmembrane protein that functions as the core subunit of mitochondrial respiratory chain complex III [50]. Mutations or functional defects in CYTB impair electron transport efficiency, disrupt oxidative phosphorylation, and ultimately lead to cellular energy supply imbalance [51]. Existing studies have confirmed that CYTB variants are closely associated with reduced exercise endurance, exercise intolerance, and lactate accumulation [52] and may also influence individual responses to training stimuli by altering mitochondrial adaptability [51,53]. Notably, exercise training itself can upregulate CYTB expression through epigenetic remodeling [54,55], induce mitochondrial biogenesis, and thereby enhance energy metabolism efficiency [56]. The significant upregulation of CYTB in Group E in this study provides a molecular explanation for the enhanced athletic performance: increased CYTB expression leads to optimized mitochondrial function, which in turn enhances energy output, forming a positive feedback loop driven by epigenetic regulation.

Our GO enrichment analysis of differentially expressed mRNAs revealed significant enrichment in processes such as nucleotide triphosphate metabolism, cellular protein degradation, and proteolytic activity involved in cellular protein degradation. KEGG enrichment analysis showed that these differentially expressed mRNAs were significantly enriched in pathways such as carbon metabolism, glycolysis/gluconeogenesis, nucleotide metabolism, and oxidative phosphorylation, consistent with findings by McGivney et al. in transcriptomic studies of muscle tissue after training in purebred horses. Gluconeogenesis, nucleotide metabolism, and oxidative phosphorylation pathways were significant, consistent with findings by McGivney et al. in a transcriptomic study of muscle tissue after training in purebred horses which showed that muscle cells prioritize the activation of mitochondrial energy metabolism and stress response pathways post-exercise [57]. Katarzyna et al. also found in a study of endurance training in Arabian horses that exercise induced the activation of metabolic and immune-related pathways [58]. These findings support the activation of energy metabolism and protein-processing-related pathways observed in our study from different perspectives.

In this study, a total of 264 differentially expressed lncRNAs were screened. Among them, the co-expressed lncRNA target genes were mainly related to transcriptional regulation, RNA metabolism, mitochondrial function, and protein degradation. These may support endurance performance in long-distance races by influencing gene transcriptional activity and energy metabolism [44]. KEGG enrichment analysis revealed that co-expressed target genes were primarily involved in pathways such as glycolysis/gluconeogenesis and microbial metabolism. Co-localized lncRNA target genes were primarily associated with carbohydrate metabolism, G protein-coupled receptor (GPCR) signaling pathways, and immune-related processes, suggesting they may influence energy utilization [59], exercise signaling [60], and immune responses [61] by regulating neighboring genes. Both co-expressed and co-localized lncRNA target genes are enriched in energy metabolism (glycolysis/gluconeogenesis, carbon metabolism) and protein processing (proteasome-related) pathways, highly consistent with mRNA differentially expressed genes, suggesting that lncRNA and mRNA may regulate horse racing performance through a synergistic network. Previous studies have shown that long non-coding RNAs play an important role in regulating exercise endurance, such as by regulating mitochondrial biogenesis [62] and muscle energy metabolism [63]. However, there is a certain degree of complementarity between the two in terms of functional characteristics: co-expression analysis emphasizes genome-wide transcriptional regulation and mitochondrial function regulation, while co-location analysis emphasizes signal transduction and immune regulation of locally adjacent genes. This suggests that lncRNAs may exert their effects on exercise performance regulation through both broad-spectrum transcriptional regulation and location-dependent gene regulation mechanisms.

This study identified 215 significantly differentially expressed circRNAs, including 104 up-regulated and 111 down-regulated circRNAs. Functional annotation revealed that they primarily participate in biological processes such as protein transport, chromosome organization, tRNA synthesis, and amino acid metabolism. KEGG pathway analysis showed enrichment in important metabolic pathways, including amino acid biosynthesis, aminoacyl-tRNA synthesis, and purine metabolism. These functions suggest that circRNAs may participate in exercise stress adaptation by regulating protein synthesis efficiency, ribosome assembly, and transport mechanisms. Liang et al. demonstrated that circLMO7 regulates muscle cell differentiation and metabolism by binding to miR-378a-3p, confirming that circRNAs can act as “sponges” for miRNAs, indirectly influencing transcription and translation processes [64]. Additionally, circRNAs are widely enriched in pathways such as aminoacyl-tRNA biosynthesis and microbial metabolism under variable environmental conditions, which aligns with the phenomenon of enhanced protein translation induced by endurance exercise. Mach et al. found in a blood multi-omics study that protein translation and metabolism-related networks exhibit systematic upregulation after exercise [65], supporting the possibility that circRNAs participate in protein synthesis and regulation in our study.

mRNA directly participates in energy metabolism and protein degradation pathways, and lncRNA influences metabolism and immune responses through transcriptional regulation and regulation of nearby genes, while circRNA may play a key role in protein synthesis, amino acid metabolism, and the maintenance of organelle function. This multi-molecular level of coordinated regulatory mechanisms can simultaneously ensure sustained energy supply, maintenance of protein homeostasis, and adaptive tissue function during prolonged high-intensity exercise in horses, thereby forming molecular differences between individuals with varying levels of athletic ability. Previous studies have shown that exercise training can significantly alter the transcriptome and non-coding RNA expression profiles of skeletal muscle [65], with lncRNAs enhancing endurance by regulating mitochondrial biogenesis and energy metabolism [59] and circRNAs being closely associated with amino acid metabolism and protein translation efficiency [66]. These findings align with the results of this study, supporting the hypothesis that mRNA–lncRNA–circRNA may form an interconnected regulatory network, jointly determining performance differences in long-distance races through multi-level metabolic and protein homeostasis regulation. However, the study’s sample size was limited and based solely on blood transcriptomic data. While this reflects systemic molecular changes, direct differences in key motor tissues such as skeletal muscle require further validation. Additionally, the functions of differentially expressed genes and non-coding RNAs primarily rely on bioinformatics predictions, lacking in vitro or in vivo experimental verification. Future studies should employ larger sample sizes and functional experiments to further elucidate their role in regulating motor performance, thereby providing more comprehensive validation and support for the hypotheses proposed in this research.

## 5. Conclusions

This study conducted whole-transcriptome sequencing on Yili horses exhibiting significant performance disparities in 5000 m races, identifying exercise-related differentially expressed genes such as EGR1, FOSB, MRPL1, LOC100049811, SIRPB2, and CYTB. It reveals that these genes and their associated mRNAs, lncRNAs, and circRNAs exert synergistic effects in energy metabolism, protein homeostasis, and muscle remodeling, potentially jointly promoting endurance and recovery capacity during long-distance races. This study provides important references for identifying candidate genes related to equine athletic performance. However, due to limited sample size and lack of functional validation, further in-depth research is required using larger samples and across multiple tissues.

## Figures and Tables

**Table 1:** Sequencing Quality and Read Length Statistics.

Raw_Reads	Clean_Reads	Error_Rate	Q20%	Q30%	GC_pct
91,493,206	89,858,044	0.01	99.12	96.24	46.09
103,900,482	100,689,662	0.01	98.97	96	46.48
94,957,100	90,466,348	0.01	98.82	95.63	47.21
78,769,258	76,519,920	0.01	98.54	96.11	49.91
86,281,582	84,191,164	0.01	98.1	94.88	49.61
78,833,206	77,067,014	0.01	98.67	96.42	49.35

## Data Availability

The data presented in this study are available upon request from the corresponding author.
